# Mental Health Benefits of a Traditional Vegetative Biofeedback Therapy Online Program during the COVID-19 Lockdown: A Controlled Trial

**DOI:** 10.3390/healthcare10101843

**Published:** 2022-09-23

**Authors:** Jorge Magalhães Rodrigues, Catarina Santos, Cristina Ventura, Jorge Machado

**Affiliations:** 1 ICBAS, School of Medicine Biomedical Sciences, University of Porto, 4050-313 Porto, Portugal; 2 IPTC—Research Department in Complementary Medicine, Portuguese Institute of Taiji and Qigong, 4470-765 Maia, Portugal; 3 CBSin—Center of BioSciences in Integrative Health, 4200-135 Porto, Portugal; 4 Health Level, ABS—Atlântico Business School, 4405-604 Vila Nova de Gaia, Portugal; 5 LABIOMEP—Porto Biomechanics Laboratory, University of Porto, 4200-450 Porto, Portugal

**Keywords:** mental health, traditional Chinese medicine, *Qigong*, *Taijiquan*, lockdown, COVID-19

## Abstract

Mandatory lockdown resulting from a pandemic may be effective against the physical impact of the virus; however, the resulting mental strains can lead to the development of several mental disturbances. *Taijiquan* and *Qigong* are considered traditional vegetative biofeedback therapies that allow the practitioner to control the functions and processes of the body through specific movements or stances, breathing techniques, and meditative exercises. This study aims to understand if these techniques can be applied as an online distance therapeutic option to reduce the psychological impact of home confinement and social distancing. Sixty-four participants were recruited and allocated to three groups. The experienced and novice *Taijiquan* and *Qigong* participants’ groups received the intervention for 8 weeks while the control group did not receive any intervention. The outcomes were psychological well-being and psychological distress levels and were assessed by the Mental Health Inventory and a written interview. The experienced *Taijiquan* and *Qigong* participants achieved significant improvements in psychological well-being and psychological distress. Novice *Taijiquan* and *Qigong* participants achieved a significant improvement in anxiety levels. Additionally, the control group showed a significant decrease in psychological well-being. This study suggests that this distance online program of *Taijiquan* and *Qigong* is feasible and may benefit the mental health of participants during a lockdown.

## 1. Introduction

The severe acute respiratory syndrome coronavirus 2 (SARS-CoV-2), which causes the “COro-naVIrus Disease 2019” (COVID-19) in humans, was first reported in December 2019 in Wuhan, China [1]. The World Health Organization (WHO) announced on 11 March 2020 that the outbreak of COVID-19 had become a pandemic [2]. Even though in May 2022, the official numbers are over 500 million confirmed cases and over 6 million deaths [3], it is estimated that excess mortality due to the COVID-19 pandemic may surpass the 18 million mark [4].

Due to the rate of infection and high demand on the health systems, drastic measures had to be taken by governments all around the world [5]. Measures such as lockdowns, telework, and social distancing would prove to be effective against the physical impact of the virus; however, it would prove to be a heavy burden on the mental health of the population [6,7,8,9,10]. Such strains can lead to the development of anxiety, depressed mood, irritability, anger, and insomnia [11], as well as long-term consequences such as alcohol abuse, PTSD, and depression [11,12].

To assist in the well-being and mental health of the population under future lockdowns, affordable and accessible tools should be studied and ready for fast delivery.

According to the literature, traditional vegetative biofeedback therapies may assist in regulating the body’s biological systems [13,14,15].

In this sense, the vegetative nervous system (usually called the autonomic nervous system) controls the involuntary functions of the body and influences the activity of internal organs, while the biofeedback process allows the patient to understand and control those functions and influence the internal biological processes [16,17].

*Taijiquan* and *Qigong* may be considered vegetative biofeedback therapies, allowing subtle changes in the body, such as relaxing certain muscles, reducing pain, and controlling microcirculation and heart rate [15]. Essentially, these techniques give us the ability to practice new ways of self-regulation [18].

These applied psychophysiological feedback techniques are patient-guided and allow the practitioner to control the functions and processes of the body through specific movements or stances, breathing techniques, and meditative exercises [18]. They are equivalent in effect and therapeutic application [19] with therapeutic *Taijiquan* considered a specific *Qigong* technique. In fact, *Qi* means “energy” and *Gong* means “work” and as a contemporary umbrella term, *Qigong* encompasses several techniques of energetic cultivation in which therapeutic *Taijiquan* perfectly fits. Taijiquan is sometimes practised with other objectives, namely as a self-defence martial art or as a performative sport; however, the processes in which its’ practice is developed are different from the therapeutic branch.

Recent studies suggest that *Taijiquan* and *Qigong* have several mental health benefits [18,19,20,21,22,23,24]. These techniques are suggested to assist in managing anxiety and depression in children and adolescents [18,24] as well as in patients with several health conditions such as breast cancer in women [20] or heart failure in elders [21], for example. The dimension of benefits seems to also reach other complex conditions such as autism [22] or ADHD [23].

Furthermore, considering that these therapies are low-cost, safe [19], require little space and are feasible in many circumstances [25], this study has the objective to understand if *Taijiquan* and *Qigong* can be applied as an online distance therapeutic option to reduce the psychological impact of home confinement and social distancing. As well, it is of utmost importance to add practical tools to the pool of available interventions to assist the population in managing emergent mental health issues.

## 2. Materials and Methods

### 2.1. Participants

Sixty-four (64) participants were recruited by resorting to online social network ads and posterior snowball sampling. Of those, fifty-four (54) were included in the study. These participants were aged between 17 and 75 years old (m = 43.43, dp = 16.158), 38.9% (21) were male and 61.1% (33) were female. 

The exclusion criteria required the participants to be absent of physical or psychiatric limitations that could prevent safe participation in the study.

The study design fulfils the ethical principles for medical research involving human subjects, according to the Declaration of Helsinki. The conducted research was analyzed and approved by the Porto University Hospital Center CHUP/School of Medicine and Biomedical Sciences ICBAS ethics committee, with the reference number 2020/CE/P012(P324/CETI/ICBAS). Informed consent was requested from all participants.

### 2.2. Group Allocation

Group allocation was not randomized due to the specific characteristics of the participants. The first phase of recruitment received applications that best-fitted group A or B (experienced and non-experienced in *Taijiquan* or *Qigong*). A second phase, best characterized by snowball sampling, aimed to recruit participants for the control group (who would not receive the intervention).

As stated above, the participants experienced in *Taijiquan* or *Qigong* were allocated to group A (GA, *n* = 13), and would receive the intervention. Participants who were not experienced in *Taijiquan* or *Qigong* but showed the desire to participate in the intervention were allocated to group B (GB, *n* = 20). Participants who agreed to participate in the study but did not desire to receive the intervention were allocated to group C (GC, *n* = 21).

**Figure 1:**
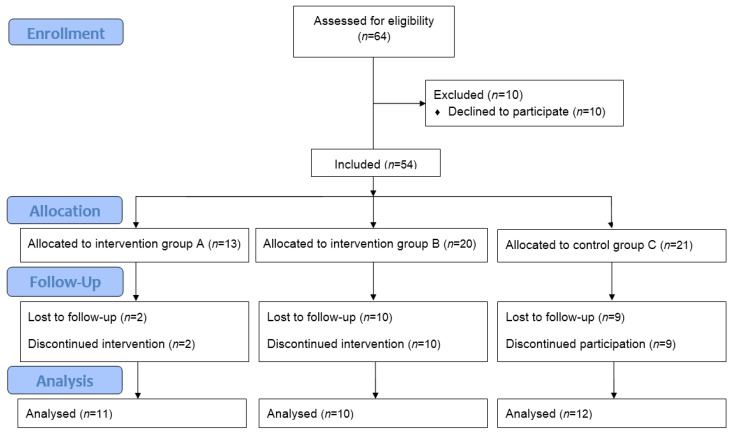
CONSORT 2010 Flow Diagram [26].

The study workflow is shown in Figure 1.

### 2.3. Intervention and Control

Intervention (groups A and B): The Shao yin vegetative biofeedback system of *Taijiquan* and *Qigong* was applied as an intervention. The system blends traditional Chinese medicine with scientific Chinese medicine models (such as the Heidelberg Model) [27,28] and has the objective of adapting traditional techniques to the modern lifestyle and technological advances. It was developed by the first author in the Portuguese Institute of Taiji and *Qigong* (*Instituto Português de Taichi e Chikung*) and has been preliminarily studied in recent years [18,23,24]. The *Qigong* techniques are mainly based on Zhan Zhuang, Ba Duan Jin, and Yi Jin Jing techniques slightly adapted to focus on the improvement oin the mental state and act on the mind, while the therapeutic *Taijiquan* techniques focused on the “5 animals” form (adapted by the first author from the 8-movements *Beijing* style *Taijiquan* form) and the 12-movements form of the “crane and the serpent” (also developed by the first author), characterized by the relatively small amount of space needed for practice, which was conveniently suited for online effective tutoring.

The online intervention program was composed of three 1-h live sessions a week and was carried out during the 8 weeks of the first lockdown decreed by the Portuguese government. The recordings of the sessions were accessible and participants were encouraged to repeat them whenever they needed them. 

Control (group C): The participants assigned to this group did not receive any intervention.

### 2.4. Outcome Measurements

The primary outcome measures were to assess the psychological well-being and the psychological distress levels of the participants.

The secondary outcome measures were positive affect, emotional ties, loss of emotional or behavioural control, anxiety, and depression levels.

All the outcomes were measured by the Mental Health Inventory (MHI) [29], adapted to the Portuguese population by Ribeiro [30]. The Mental Health Inventory–38 (MHI–38) is a 38-item self-report tool designed to measure general psychological distress (anxiety, depression, loss of emotional or behavioural control) and well-being (positive affect, emotional ties). Each question is rated on a specific 6-choice frequency rating.

Primary and secondary outcome measures were assessed in two moments. The pre-intervention assessment was conducted at the beginning of the experimental phase, and a post-intervention assessment after 8 weeks.

At the end of the experimental phase, participants in groups A and B were asked to answer a simple written interview to assess qualitative elements to support quantitative data discussion.

### 2.5. Statistical Analysis

The statistical analysis was conducted using IBM SPSS statistics 25 software version 27 (Armonk, NY, USA). The non-parametric Kruskal–Wallis H-test was used to compare outcome measure scores between groups, and the Wilcoxon signed-rank test was used to assess differences between scores in different moments of evaluation. The effect was considered statistically significant when the *p*-value was less than 0.05 (95% confidence interval).

### 2.6. Qualitative Analysis

Data analysis was conducted according to the principles of thematic analysis. A codebook methodology for qualitative analysis was chosen, as proposed by Clarke and Braun [31], which is a mixed big Q and small q qualitative research approach. The reasoning for this type of work is predominantly qualitative; however, the initial themes of the analysis can be changed during the procedure of the analysis, approaching it with a more flexible and reflexive approach [32].

Two researchers coded the interviews and summarized the data. A registry unit was chosen to represent each sub-theme. When there was a disagreement, a third researcher would settle the issue.

## 3. Results

### 3.1. Quantitative Results

#### 3.1.1. Pre-Intervention

The pre-intervention comparison between groups showed no significant differences in “psychological well-being”, H(2) = 0.260, P = 0.878. As well, no differences were found for “positive affect” H(2) = 0.324, P = 0.851, and “emotional ties” H(2) = 0.081, P = 0.960.

The absence of significant differences was also found for “psychological distress” H(2) = 1.101, P = 0.577, and the dimensions “loss of emotional or behavioural control” H(2) = 0.090, P = 0.956, “anxiety” H(2) = 2.018, P = 0.365, and “depression” H(2) = 1.327, P = 0.515.

#### 3.1.2. Post-Intervention

The post-intervention comparison between groups showed some statistically significant differences (Table 1), namely in the psychological well-being dimension and specifically in Positive Affect.

Pairwise comparisons using Dunn’s test revealed that on both occasions the differences were only significant between group A and group C. Table 2 presents these results, with *p*-values adjusted for Bonferroni correction.

- Group A

The Wilcoxon signed-rank test revealed that the “psychological well-being” scores were significantly higher after the intervention (Md = 62) compared to before (Md = 55), Z = 2.272, P = 0.023, with a medium effect size (r = 0.49).

It was also observed that there was a statistically significant improvement in “positive affect” scores following participation in the program, Z = 2.316, P = 0.021, with a medium effect size (r = 0.49). The median score on the “positive affect” scale improved from the pre-intervention (Md = 44) to the post-intervention (Md = 51).

Regarding the “psychological distress” scores, they were significantly lower after the intervention (Md = 54) compared to before (Md = 63), Z = −2.002, P = 0.045, with a medium effect size (r = 0.43).

As well, “depression” scores were higher before applying the online program (Md = 13) in comparison with post-program scores (Md = 8), Z = −2.507, P = 0.012, and large effect size (r = 0.53).

No other dimensions achieved statistically significant changes between the two moments.

- Group B

In group B, the Wilcoxon signed-rank test only revealed a significant difference for the “anxiety” dimension (Z = −2.040, P = 0.041). A decrease from pre-intervention (Md = 33) to post-intervention scores (Md = 28) were observed with a medium effect size (r = 0.46).

- Group C

Concerning participants of group C, Wilcoxon’s signed-rank test revealed significant differences in “psychological well-being” (Z = −2.476, P = 0.013) and its “positive affect” dimension (Z = −2.497, P = 0.013), both with a large effect size (r = 0.51).

The median score on the “psychological well-being” scale decreased from the beginning (Md = 57) to the end of the study (Md = 47). Similarly, the median score on the “positive affect” scale decreased from the baseline (Md = 46.5) to the final moment (Md = 36.5).

### 3.2. Qualitative Results

The three major themes were: “Main sensations and benefits elicited by the therapy” concerning the perceptions of the participants about how they feel when performing Taiji and/or *Qigong*; “Main impact of lockdown on mental health” explores how the lockdown impacted the mental health of the participants; and “Main benefits of the therapy for mental health during lockdown” considers the perceived benefits of the online program in the mental health of the participants.

For each of the themes, several sub-themes were identified. The sub-themes and respective main registry units can be observed in Table 3. The number of references is also shown and represents the number of times the sub-theme was identified in the interviews.

## 4. Discussion

According to Abbott and Lavretsky [33], *Taijiquan* and *Qigong* are evidence-based therapies that improve health-related quality of life and can be effective in reducing depressive symptoms, anxiety, and stress. Despite the constant need for improving methodology and research quality, several studies indicate that the benefits of these techniques may indeed be relevant in several contexts of the mental health field [18,22,23,24,33,34,35,36,37]. Instead of focusing on the treatment of mental disorders, our study focused on the prevention capacities of these techniques, following the therapeutic application as an applied psychophysiological feedback technique. According to Posadzki, et al. [38], *Qigong* enhances the mind’s self-regulatory processes and prevents mental health disorders, which is also suggested by our results.

In our study, the psychological well-being dimension showed significant differences in post-intervention assessment between the group of experienced *Taijiquan* and *Qigong* participants and the control group. Actually, the pre- post-intervention comparison showed significant improvements for the experienced *Taijiquan* and *Qigong* participants’ group, while the control group participants demonstrated deterioration of these levels. The novice group did not show significant changes, suggesting that intervention may regulate the levels of psychological well-being, acting as a preventative option for non-experienced *Taijiquan* and *Qigong* participants.

Moreover, these results are also suggested by the study of Robles et al. [39], which assessed a virtual *Taijiquan* and *Qigong* intervention, concluding that it may be able to improve psychological well-being in university older employees. Participants in the intervention groups also seem to express this sentiment when systematically expressing statements, such as the practice helps “(…) achieve some serenity, mental and spiritual balance…” and “(…) feel a greater balance, psychological, physical and even spiritual. Sometimes, at the end of the practice, I feel a great satisfaction”. Besides increased positive mood states and reduced anxiety, the study of Johansson and Hassmén [40] also supported the sense that these practices induce a sense of pleasure. In this line of thought, and according to our results, these positive changes in psychological well-being are mainly due to the variation in the levels of positive affect. Positive affect is a characteristic that may explain how much people experience positive sensations, emotions, or sentiments [41]. Higher levels of positive affectivity result in healthier coping styles, more enthusiasm, energy, and confidence, it increases problem-solving capacities, longevity, improves sleep, and decreases stress hormones [42,43].

During the COVID-19 lockdown, participants exposed to the intervention showed increased (experienced group) or stable (novice group) levels of positive affect, while non-participants seem to show a decrease in those levels.

It is also proposed by Ashby and Isen [41] that positive affect levels influence the consolidation of long-term memories, working memory, and creative problem-solving. Accordingly, participants in this study expressed this idea by stating that they feel an “Improvement of concentration and memory, which is reflected in the clarity of reasoning, mood and disposition”. 

Furthermore, the improvement in cognitive processes was also observed in the study of Li, et al. [44] which suggested that *Taijiquan* delivered remotely is feasible, well-accepted, and safe when designed as a cognitively enhanced training intervention for older adults with mild cognitive impairment.

As expected as a consequence of the COVID-19 pandemic, psychological distress would increase [45,46,47,48]. Agreeing participants in this study stated that “(…) preoccupation with employment/economic state also negatively affected my mental state.” and that “the break from the routine and the need to create a new routine never thought of before, causes discomfort and anxiety”. The lockdown left the sense of “being unnatural and forced” which would lead “to depressive emotional states and a sense of desperate loneliness…”.

Regarding this topic, the results of our study show that this online program can attenuate the psychological impact of the pandemic since improvements in depression symptoms were observed in the experienced *Taijiquan* and *Qigong* participants’ group and improvements in anxiety were found in the novice group. On the discourse of several participants, the program helped “… by improving anxiety, it also improves good thoughts, releases endorphins, causes relaxation, which helped me sleep”, achieving an internal balance when stating “overall, I feel that with Taiji and *Qigong* I’m back to doing a more balanced synchronization with myself” and satisfaction by “…a feeling of continuity and a rhythm of normality and satisfaction in times of uncertainty, allowing an evolution and growth of knowledge…”. As well, the *Taijiquan* online program of Oh et al. [49], also conducted during the COVID-19 pandemic, resulted in an increased overall satisfaction and quality of life for participants.

This quality of life extends from psychological well-being to physical well-being when we observe that participants state that they feel a “(…) sense of tranquility and relaxation, clear thinking, combined with respiratory and muscular improvements, physical well-being, flexibility and balance…”. As well, it is stated that improvements translate into “better balance and flexibility, activation of blood circulation, endurance and breath control.” Actually, the physiologic mechanisms that explain these benefits may be the decrease in the sympathetic output [50,51] or even the modulation of both the autonomic divisions of the nervous system [52,53]. In addition, it can also increase endorphin blood levels [54], and reduce the levels of inflammatory markers [55], adrenocorticotropic hormone [54], and cortisol [18,56,57].

Overall, it is important to note that unusual times (such as those of a pandemic) require the use of facilitating technologies that we have at our disposal, to improve reach and access by the population.

In agreement with other studies [39,44,49,58], we claim that these distance-delivered interventions are feasible, and welcomed by people; however, there is an emergent necessity to develop guidelines for *Taijiquan* and *Qigong* training and education for distance therapeutic application.

Specifically related to this study, our main limitation relates to the number of participants. A higher number of participants would allow a more robust methodological design. We also suggested that the use of a placebo group would also improve the results’ discussion and the internal validity of the study. Another limitation is the dropout rate which, in this study, was around 40%. This seems to happen because it is difficult to keep participants engaged in some distance interventions without therapists or other in-person support [59,60]. In the experimental groups, nearly 85% of the dropouts were from the “novice” group (50% dropout rate) which may suggest that people who are familiar with the techniques may have stronger motivation to remain in this specific kind of distance model intervention. Difficulty in obtaining the reason for discontinuing the intervention was also observed as none of the dropouts gave feedback about their reason for leaving the experiment. Finally, the random group allocation model should be applied to reduce possible participants’ preconceived biases about the intervention.

## 5. Conclusions

The results of this study suggest that *Taijiquan* and *Qigong* can be applied as a traditional vegetative biofeedback therapy by means of a distance online program. Employed as such, it may be able to improve psychological well-being and reduce psychological distress during stressful life events that require social distancing, such as a pandemic. 

## Figures and Tables

**Table 1:** Post-intervention Kruskal–Wallis test results for the mental health dimensions.

	Kruskal–Wallis Test	
Dimensions	H	P
Psychological Well-being	7.711	**0.021**
Positive Affect	6.819	**0.033**
Emotional ties	2.074	0.355
Psychological Distress	3.451	0.178
Loss of emotional or behavioural control	2.357	0.308
Anxiety	2.423	0.298
Depression	4.969	0.083

H: Kruskal–Wallis test statistic. P: probability value.

**Table 2:** Pairwise comparison results in Psychological Well-being and Positive Affect.

	Dunn’s Test for Pairwise Comparisons		
	A–B	A–C	B–C
Dimensions	P	P	P
Psychological Well-being	0.468	**0.027**	0.821
Positive Affect	0.484	**0.016**	0.604

A–B: group A and B pairwise comparison. A–C: group A and C pairwise comparison. B–C: group B and C pairwise comparison. P: probability value.

**Table 3:** Themes, sub-themes and main registry units retrieved from the qualitative analysis of the interviews.

Theme	Sub-Theme	Main Registry Units	References
Main sensations and benefits elicited by the program	Mental well-being	“(…) achieve some serenity, mental and spiritual balance…”	[21]
	Improved body dynamics	“Better balance and flexibility, activation of blood circulation, endurance and breath control.”	[14]
	Improvement in cognitive processes	“Improvement of concentration and memory, which is reflected in the clarity of reasoning, mood and disposition”	[7]
	Integrated balance	“(…) I feel a greater balance, psychological, physical and even spiritual. Sometimes, at the end of the practice, I feel great satisfaction.”	[4]
	Breathing improvements	“Calmer, greater body flexibility and breathing improvements (…)”	[3]
Main impact of lockdown on mental health	Alteration of the emotional state	“The break from the routine and the need to create a new routine never thought of before, causes discomfort and anxiety.”“ (…) preoccupation with employment/economic state also negatively affected my mental state.”	[9]
	Stress, loneliness, and imprisonment sensation	“Being unnatural and forced lead me to depressive emotional states and a sense of desperate loneliness…”	[6]
	Lack of motivation	“(…) I had days when I felt irritated with people online because of problems, because of work that was not exactly tiring but continuous, or because of lack of motivation to do things”	[2]
Main benefits of the program for mental health during lockdown	Tranquillity	“(…) sense of tranquillity and relaxation, clear thinking, combined with respiratory and muscular improvements, physical well-being, flexibility and balance…”	[5]
	Relaxation	“… By improving anxiety, it also improves good thoughts, releases endorphins, and causes relaxation, which helped me sleep.”	[4]
	Internal balance	“Overall, I feel that with Taichi and Chikung I’m back to doing a more balanced synchronization with myself.”	[3]
	Satisfaction	“… a feeling of continuity and a rhythm of normality and satisfaction in times of uncertainty, allowing an evolution and growth of knowledge…”	[3]

## Data Availability

Not applicable.

## References

[1] ProMED Undiagnosed Pneumonia—China (Hubei): Request for Information. https://scholar.harvard.edu/files/kleelerner/files/20191230_promed_-_undiagnosed_pneumonia_-_china_hu-_rfi_archive_number-_20191230.6864153.pdf.

[2] Asselah T., Durantel D., Pasmant E., Lau G., Schinazi R.F. (2021). COVID-19: Discovery, diagnostics and drug development. J. Hepatol..

[3] World Health Organization (2022). Weekly Epidemiological Update on COVID-19—4 May 2022.

[4] Wang H., Paulson K.R., Pease S.A., Watson S., Comfort H., Zheng P., Aravkin A.Y., Bisignano C., Barber R.M., Alam T. (2022). Estimating excess mortality due to the COVID-19 pandemic: A systematic analysis of COVID-19-related mortality, 2020–2021. Lancet.

[5] Wang C., Horby P.W., Hayden F.G., Gao G.F. (2020). A novel coronavirus outbreak of global health concern. Lancet.

[6] Thakur K., Kumar N., Sharma N. (2020). Effect of the pandemic and lockdown on mental health of children. Indian J. Pediatr..

[7] Adams-Prassl A., Boneva T., Golin M., Rauh C. (2020). The impact of the coronavirus lockdown on mental health: Evidence from the US. Econ. Policy.

[8] Jacques-Aviñó C., López-Jiménez T., Medina-Perucha L., de Bont J., Gonçalves A.Q., Duarte-Salles T., Berenguera A. (2020). Gender-based approach on the social impact and mental health in Spain during COVID-19 lockdown: A cross-sectional study. BMJ Open.

[9] Dawes J., May T., McKinlay A., Fancourt D., Burton A. (2021). Impact of the COVID-19 pandemic on the mental health and wellbeing of parents with young children: A qualitative interview study. BMC Psychol..

[10] Rubin G.J., Wessely S. (2020). The psychological effects of quarantining a city. BMJ.

[11] Brooks S.K., Webster R.K., Smith L.E., Woodland L., Wessely S., Greenberg N., Rubin G.J. (2020). The psychological impact of quarantine and how to reduce it: Rapid review of the evidence. Lancet.

[12] Wu P., Liu X.H., Fang Y.Y., Fan B., Fuller C.J., Guan Z.Q., Yao Z.L., Kong J.H., Lu J., Litvak I.J. (2008). Alcohol Abuse/Dependence Symptoms Among Hospital Employees Exposed to a SARS Outbreak. Alcohol Alcohol..

[13] Greten H.J., Xia Y., Ding G., Wu G.-C. (2013). Chinese Medicine as a Model of System Biology: Diagnosis as the Foundation of Acupoint Selection. Current Research in Acupuncture.

[14] Greten H.J. (2014). Qi Gong: Scientific Chinese Medicine—The Heidelberg Model.

[15] Matos L.C., Sousa C.M., Goncalves M., Gabriel J., Machado J., Greten H.J. (2015). Qigong as a Traditional Vegetative Biofeedback Therapy: Long-Term Conditioning of Physiological Mind-Body Effects. BioMed Res. Int..

[16] Zhang Z., Wu H., Wang W., Wang B. A smartphone based respiratory biofeedback system. Proceedings of the 2010 3rd International Conference on Biomedical Engineering and Informatics.

[17] Giggins O.M., Persson U.M., Caulfield B. (2013). Biofeedback in rehabilitation. J. Neuroeng. Rehabil..

[18] Rodrigues J.M., Matos L.C., Francisco N., Dias A., Azevedo J., Machado J. (2021). Assessment of Qigong Effects on Anxiety of High-school Students: A Randomized Controlled Trial. Adv. Mind. Body Med..

[19] Jahnke R., Larkey L., Rogers C., Etnier J., Lin F. (2010). A comprehensive review of health benefits of qigong and tai chi. Am. J. Health Promot..

[20] Meng T., Hu S.F., Cheng Y.Q., Ye M.N., Wang B., Wu J.J., Chen H.F. (2021). Qigong for women with breast cancer: An updated systematic review and meta-analysis. Complement. Ther. Med..

[21] Jiang W., Liao S., Chen X., Lundborg C.S., Marrone G., Wen Z., Lu W. (2021). TaiChi and Qigong for Depressive Symptoms in Patients with Chronic Heart Failure: A Systematic Review with Meta-Analysis. Evid. Based Complement. Alternat. Med..

[22] Rodrigues J.M., Mestre M., Fredes L.I. (2019). Qigong in the treatment of children with autism spectrum disorder: A systematic review. J. Integr. Med..

[23] Rodrigues J.M., Mestre M., Matos L.C., Machado J.P. (2019). Effects of taijiquan and qigong practice over behavioural disorders in school-age children: A pilot study. J. Bodyw. Mov. Ther..

[24] Rodrigues J.M., Lopes L., Goncalves M., Machado J.P. (2021). Taijiquan and qigong as a mindfulness cognitive-behavioural based therapy on the treatment of cothymia in school-age children—A preliminary study. J. Bodyw. Mov. Ther..

[25] Sang X., Menhas R., Saqib Z.A., Mahmood S., Weng Y., Khurshid S., Iqbal W., Shahzad B. (2021). The Psychological Impacts of COVID-19 Home Confinement and Physical Activity: A Structural Equation Model Analysis. Front. Psychol..

[26] Schulz K.F., Altman D.G., Moher D. (2011). CONSORT 2010 statement: Updated guidelines for reporting parallel group randomised trials. Int. J. Surg..

[27] Greten H.J. (2011). Chinese medicine as vegetative systems biology. Part I: Therapeutic methods. HNO.

[28] Matos L.C., Machado J.P., Monteiro F.J., Greten H.J. (2021). Understanding Traditional Chinese Medicine Therapeutics: An Overview of the Basics and Clinical Applications. Healthcare.

[29] Veit C.T., Ware J.E. (1983). The structure of psychological distress and well-being in general populations. J. Consult. Clin. Psychol..

[30] Ribeiro J.L.P. (2001). Mental Health Inventory: Um estudo de adaptação à população portuguesa. Psicol. Saúde E Doenças.

[31] Clarke V., Braun V. (2017). Thematic analysis. J. Posit. Psychol..

[32] Souza L.K.d. (2019). Pesquisa com análise qualitativa de dados: Conhecendo a Análise Temática. Arq. Bras. Psicol..

[33] Abbott R., Lavretsky H. (2013). Tai Chi and Qigong for the Treatment and Prevention of Mental Disorders. Psychiatr. Clin. N. Am..

[34] Chang P.-S., Knobf T., Oh B., Funk M. (2019). Physical and Psychological Health Outcomes of Qigong Exercise in Older Adults: A Systematic Review and Meta-Analysis. Am. J. Chin. Med..

[35] Niles B.L., Reid K.F., Whitworth J.W., Alligood E., Williston S.K., Grossman D.H., McQuade M.M., Mori D.L. (2022). Tai Chi and Qigong for trauma exposed populations: A systematic review. Ment. Health Phys. Act..

[36] Chen C.-H., Hung K.-S., Chung Y.-C., Yeh M.-L. (2019). Mind–body interactive qigong improves physical and mental aspects of quality of life in inpatients with stroke: A randomized control study. Eur. J. Cardiovasc. Nurs..

[37] Tong H., Liu Y., Zhu Y., Zhang B., Hu J. (2019). The therapeutic effects of qigong in patients with chronic obstructive pulmonary disease in the stable stage: A meta-analysis. BMC Complement. Altern. Med..

[38] Posadzki P., Parekh S., Glass N. (2010). Yoga and qigong in the psychological prevention of mental health disorders: A conceptual synthesis. Chin. J. Integr. Med..

[39] Robles D., Yang Y., Wager M., Kang P., Sokan A., Yuan N., Chen Z. Benefits of virtual Tai Chi and Qigong intervention on sleep quality and wellbeing among university older employees. Proceedings of the 2nd International Conference on Chinese Health Practices-Tai Chi.

[40] Johansson M., Hassmén P. (2008). Acute Psychological Responses to Qigong Exercise of Varying Durations. Am. J. Chin. Med..

[41] Ashby F.G., Isen A.M. (1999). A neuropsychological theory of positive affect and its influence on cognition. Psychol. Rev..

[42] Li Y.I., Starr L.R., Hershenberg R. (2017). Responses to Positive Affect in Daily Life: Positive Rumination and Dampening Moderate the Association Between Daily Events and Depressive Symptoms. J. Psychopathol. Behav. Assess..

[43] Paterson T.S.E., Yeung S.E., Thornton W.L. (2016). Positive affect predicts everyday problem-solving ability in older adults. Aging Ment. Health.

[44] Li F., Harmer P., Fitzgerald K., Winters-Stone K. (2022). A cognitively enhanced online Tai Ji Quan training intervention for community-dwelling older adults with mild cognitive impairment: A feasibility trial. BMC Geriatr..

[45] Romero E., López-Romero L., Domínguez-Álvarez B., Villar P., Gómez-Fraguela J.A. (2020). Testing the Effects of COVID-19 Confinement in Spanish Children: The Role of Parents’ Distress, Emotional Problems and Specific Parenting. Int. J. Environ. Res. Public Health.

[46] Husky M.M., Kovess-Masfety V., Swendsen J.D. (2020). Stress and anxiety among university students in France during Covid-19 mandatory confinement. Compr. Psychiatry.

[47] Lal A., Sanaullah A., Saleem M.K.M., Ahmed N., Maqsood A., Ahmed N. (2020). Psychological Distress among Adults in Home Confinement in the Midst of COVID-19 Outbreak. Eur. J. Dent..

[48] Odriozola-González P., Planchuelo-Gómez Á., Irurtia M.J., de Luis-García R. (2020). Psychological symptoms of the outbreak of the COVID-19 confinement in Spain. J. Health Psychol..

[49] Oh B., Van Der Saag D., Morgia M., Carroll S., Boyle F., Back M., Lamoury G. (2020). An Innovative Tai Chi and Qigong Telehealth Service in Supportive Cancer Care During the COVID-19 Pandemic and Beyond. Am. J. Lifestyle Med..

[50] Irwin M.R., Olmstead R., Motivala S.J. (2008). Improving sleep quality in older adults with moderate sleep complaints: A randomized controlled trial of Tai Chi Chih. Sleep.

[51] Motivala S.J., Sollers J., Thayer J., Irwin M.R. (2006). Tai Chi Chih acutely decreases sympathetic nervous system activity in older adults. J. Gerontol. Ser. A Biol. Sci. Med. Sci..

[52] Rodrigues J.M., Lopes L., Goncalves M., Machado J.P. Health Benefits of Taijiquan and Qigong: Participants’ Perception. Proceedings of the International Cappadocia Scientific Research Congress.

[53] Rodrigues J.M., Lopes L., Goncalves M., Machado J.P. (2022). Perceived health benefits of taijiquan and qigong. Altern. Ther. Health Med..

[54] Ryu H., Lee H.S., Shin Y.S., Chung S.M., Lee M.S., Kim H.M., Chung H.T. (1996). Acute effect of qigong training on stress hormonal levels in man. Am. J. Chin. Med..

[55] Lavretsky H., Alstein L.L., Olmstead R.E., Ercoli L.M., Riparetti-Brown M., Cyr N.S., Irwin M.R. (2011). Complementary use of tai chi chih augments escitalopram treatment of geriatric depression: A randomized controlled trial. Am. J. Geriatr. Psychiatry.

[56] Lee M.S., Kang C.W., Lim H.J., Lee M.S. (2004). Effects of Qi-training on anxiety and plasma concentrations of cortisol, ACTH, and aldosterone: A randomized placebo-controlled pilot study. Stress Health.

[57] Lee M.S., Lee M.S., Kim H.J., Moon S.R. (2003). Qigong reduced blood pressure and catecholamine levels of patients with essential hypertension. Int. J. Neurosci..

[58] Sawyer L.M., Brown L.M., Lensing S.Y., McFadden D., Bopp M.M., Ferrier I., Sullivan D.H. (2022). Rapid conversion of Tai Chi classes from face-to-face to virtual during the COVID-19 pandemic: A quality improvement project. Nurs. Forum.

[59] Pratap A., Renn B.N., Volponi J., Mooney S.D., Gazzaley A., Arean P.A., Anguera J.A. (2018). Using Mobile Apps to Assess and Treat Depression in Hispanic and Latino Populations: Fully Remote Randomized Clinical Trial. J. Med. Internet Res..

[60] Rodrigues J.M., Oliveira F., Ribeiro C.P., Santos R.C., António M., Ricardo Q. (2022). Mobile Mental Health for Depression Assistance. Digital Therapies in Psychosocial Rehabilitation and Mental Health.

